# Transfer Learning-Based Quantification of Mitotic Activity and Its Prognostic and Molecular Associations in Lung Squamous Cell Carcinoma

**DOI:** 10.3390/cancers18193098

**Published:** 2026-09-23

**Authors:** Gulcin Yilmaz Gunes, Mehmet Akif Cifci

**Affiliations:** 1 Department of Pulmonology, Bandırma Training and Research Hospital, Bandırma 10200, Türkiye; drgulcinyilmaz@gmail.com; 2 Institute of Research and Development, Duy Tan University, Da Nang 550000, Vietnam; 3 School of Engineering and Technology, Duy Tan University, Da Nang 550000, Vietnam; 4 Department of Computer Engineering, Bandırma Onyedi Eylül University, Balıkesir 10200, Türkiye; 5 Engineering and Informatics Department, Klaipėdos Valstybinė Kolegija/Higher Education Institution, 92294 Klaipeda, Lithuania

**Keywords:** lung squamous cell carcinoma, mitosis, transfer learning, computational pathology, tumor-area mitosis proportion, deep learning

## Abstract

**Background:** Automated assessment of mitotic activity may provide a reproducible marker of tumor proliferation, but computational mitosis recognition remains challenging because of cross-domain variability. We developed a transfer-learning framework to quantify mitotic activity in lung squamous cell carcinoma (LUSC) and investigated its clinical and molecular associations. **Methods:** A ResNet-34 classifier was trained using MIDOG source-domain data and adapted to TCGA-LUSC diagnostic whole-slide images. The target cohort included 478 patients and 512 whole-slide images. Tumor regions and mitotic figures were annotated by two pathologists, and 128 × 128-pixel patches at 40× resolution were analyzed. After identifying patient overlap in the initial patch-level partition, we implemented patient-level stratified five-fold cross-validation to obtain leakage-mitigated performance estimates and out-of-fold predictions. Tumor-area mitosis proportion (TAMP) was defined as the proportion of evaluable tumor-region patches classified as mitosis-positive. Prognostic associations were evaluated using multivariable Cox proportional-hazards regression adjusted for age, sex, and pathological stage. **Results:** The classifier achieved an AUC of 0.972 (95% CI, 0.965–0.980) on the internal MIDOG test set. Direct application to TCGA-LUSC yielded an AUC of 0.81, whereas target-domain fine-tuning produced an initial patch-level AUC of 0.93. Patient tracing identified overlap in the original partitions, with 142 patients (29.7%) contributing patches to multiple subsets, including 67 patients (14.0%) represented in both training and test sets. Leakage-mitigated patient-level cross-validation yielded a mean AUC of 0.88 (95% CI, 0.86–0.90) and PR-AUC of 0.24. A patient-disjoint WSI holdout evaluation yielded an AUC of 0.87 (95% CI, 0.84–0.90) and PR-AUC of 0.22. High TAMP was associated with poorer overall survival (log-rank p=0.017; HR = 1.73, 95% CI, 1.12–2.68) and remained significant after adjustment for age, sex, and pathological stage (adjusted HR = 1.62, 95% CI, 1.03–2.54; p=0.036). High-TAMP tumors showed enrichment of proliferation- and growth-related transcriptional programs, whereas immune, genomic, and predicted drug-response associations were modest and hypothesis-generating. **Conclusions:** TAMP represents an image-derived measure of mitotic activity associated with survival and molecular characteristics in TCGA-LUSC. The findings support further investigation of computational mitotic phenotyping, but external multicenter validation and prospective clinical assessment are required before clinical translation.

## Simple Summary

Mitotic activity reflects how actively tumor cells are dividing and may provide information about cancer aggressiveness. However, manual assessment is time-consuming and may vary among pathologists. We developed a computer-based method to quantify mitotic activity in digitized tissue images from patients with lung squamous cell carcinoma. We evaluated the method using patient-separated data and examined whether the resulting measure was associated with survival and molecular tumor characteristics. We found that patients with higher predicted mitotic activity had poorer overall survival. This association remained significant after accounting for age, sex, and tumor stage. The results support automated mitotic assessment as a reproducible method for investigating tumor behavior and prognosis. Independent patient cohorts and prospective clinical studies are required before this method can support clinical decision-making.

## 1. Introduction

Lung cancer remains the leading cause of cancer mortality worldwide. Global Cancer Statistics 2024 estimated approximately 2.6 million new lung cancer cases and 1.9 million deaths in 2024, making lung cancer both the most frequently diagnosed malignancy and the largest contributor to global cancer mortality, accounting for approximately 19.1% of cancer deaths among an estimated 20.6 million new cancer cases and 9.8 million cancer deaths worldwide [1]. This burden has remained consistently high over the past decade of global surveillance [2]. Lung squamous cell carcinoma (LUSC), a major subtype of non-small cell lung cancer, accounts for approximately 25–30% of lung cancer cases and typically arises from the bronchial epithelium of the central airways [3,4]. LUSC is strongly associated with smoking and is characterized by frequent genomic alterations involving *TP53*, *CDKN2A*, *PIK3CA*, and other regulators of cell-cycle control and tumor growth [5]. Compared with lung adenocarcinoma, therapeutic options based on actionable molecular alterations remain more limited, emphasizing the need for additional biomarkers that can characterize tumor aggressiveness and support prognostic stratification [6].

Uncontrolled cellular proliferation is a fundamental hallmark of cancer [7,8]. Histologically, mitotic figures provide a direct morphological indication of active cell division and are widely used as indicators of tumor proliferation and biological aggressiveness [9]. Mitotic activity already contributes to established grading systems in several malignancies, including breast cancer and pulmonary neuroendocrine neoplasms [10,11]. In LUSC, increased mitotic activity has also been associated with aggressive pathological characteristics and cell-cycle dysregulation, although mitotic assessment has not been incorporated into a standardized quantitative framework for routine prognostic evaluation [12].

Conventional mitotic counting relies on manual microscopic examination and is therefore time-consuming and susceptible to interobserver variability [13]. Reliable identification is further complicated by morphological mimics such as apoptotic bodies, hyperchromatic nuclei, and tissue artifacts [14,15]. These limitations are particularly relevant in whole-slide imaging, where exhaustive evaluation of large tumor regions is impractical. A reproducible computational approach capable of quantifying mitotic activity across extensive tumor areas may therefore provide a more standardized representation of proliferative activity.

Recent advances in computational pathology and deep learning have enabled automated analysis of histopathological whole-slide images [16]. Convolutional neural networks have demonstrated strong performance for mitotic figure recognition, while benchmark initiatives such as the Mitosis Domain Generalization (MIDOG) challenges have highlighted the importance of robustness across scanners, laboratories, staining conditions, and tissue domains [17]. Nevertheless, domain shift remains a major obstacle when models trained on one tumor type are transferred to another. Transfer-learning strategies provide a practical approach to this problem by retaining broadly informative morphological representations while adapting higher-level features to the target tissue [18].

Beyond automated detection, computational pathology provides an opportunity to connect quantitative morphological phenotypes with clinical and molecular characteristics. TCGA-LUSC combines diagnostic whole-slide images with clinical, genomic, and transcriptomic information, enabling systematic investigation of relationships between histological features and tumor biology [5]. However, the association between computationally quantified mitotic activity and survival, tumor microenvironment composition, genomic instability, pathway activity, predicted drug response, and transcriptional regulation in LUSC remains insufficiently characterized.

Accordingly, this study developed a transfer-learning framework pre-trained on MIDOG data and adapted to TCGA-LUSC for patch-level mitosis classification. We define tumor-area mitosis proportion (TAMP) as a patch-derived computational measure of mitotic activity within pathologist-defined tumor regions. TAMP was subsequently examined in relation to overall survival, tumor microenvironment characteristics, genomic alterations, pathway enrichment, predicted drug sensitivity, and inferred transcription-factor activity. By integrating computational histopathology with clinical and multi-omics data, this study aims to characterize the biological and prognostic associations of mitotic activity in LUSC and provide a foundation for further patient-level validation.

The principal contribution of this study is the integration of a patient-independent, image-derived mitotic phenotype with clinical outcome and multi-omics characteristics in LUSC rather than the development of a novel neural-network architecture. This contribution has four components. First, we transfer mitotic-morphology representations learned from MIDOG to LUSC whole-slide image patches using a two-stage transfer-learning strategy. Second, we evaluate target-domain performance under patient-independent conditions, using patient-level stratified five-fold cross-validation to address overlap identified in the initial patch-level partition. Third, we derive tumor-area mitosis proportion (TAMP) from patient-level out-of-fold predictions and evaluate its association with overall survival. Fourth, we integrate TAMP with tumor microenvironment composition, genomic characteristics, transcriptomic pathway enrichment, inferred transcription-factor activity, and predicted drug-response profiles to provide a broader molecular characterization of highly mitotic LUSC. Together, these contributions extend automated mitosis analysis beyond patch-level classification toward an integrated, patient-independent computational pathology framework.

## 2. Literature Review

Mitotic activity is a well-established histopathological marker of tumor proliferation and aggressiveness. In several malignancies, mitotic count is incorporated directly into diagnostic or grading systems because it provides a morphological measure of active cell division. In breast cancer, mitotic activity contributes to the Nottingham histological grade [19]. In pulmonary neuroendocrine neoplasms, mitotic count is a key criterion for distinguishing typical carcinoid, atypical carcinoid, and high-grade neuroendocrine carcinomas. Increased mitotic activity has also been associated with aggressive histological features, higher proliferative capacity, and adverse clinical behavior in non-small cell lung cancer, including lung squamous cell carcinoma (LUSC) [20]. However, a standardized and reproducible framework for quantitative mitotic assessment in LUSC has not yet been established [21].

Manual mitotic counting remains challenging in routine histopathology. The process is labor-intensive, depends on the selection of representative high-power fields, and is affected by interobserver variability [17]. Reliable identification is further complicated by morphological mimics such as apoptotic bodies, hyperchromatic nuclei, pyknotic cells, and tissue-processing artifacts [22]. These limitations have motivated the development of automated image-analysis methods capable of detecting mitotic figures consistently across hematoxylin-and-eosin-stained whole-slide images [23].

Early computational approaches relied primarily on handcrafted morphological, texture, and color features combined with conventional machine-learning classifiers [24]. The emergence of deep convolutional neural networks substantially improved automated mitosis recognition by enabling direct learning of discriminative morphological representations from image data [15]. Architectures based on residual networks, DenseNet, EfficientNet, and related convolutional backbones have demonstrated strong performance for patch-level mitosis classification [25,26,27]. Nevertheless, models trained under controlled conditions frequently experience performance degradation when applied to images acquired using different scanners, staining protocols, laboratories, or tissue types [28]. This domain-shift problem remains one of the major barriers to robust computational pathology systems [29].

To improve detection accuracy and reduce false-positive predictions, subsequent studies increasingly adopted multi-stage architectures. These approaches commonly separate candidate generation from final mitosis classification, allowing a sensitive first stage to identify potential mitotic regions followed by a more discriminative refinement stage [30]. Detection frameworks based on Mask R-CNN and related region-based models have shown promising performance in histological mitosis detection [31]. RetinaNet-based approaches have also been investigated for direct detection of mitotic figures in whole-slide imagery [32]. More recent systems have combined rapid candidate segmentation with high-capacity classification networks, improving both computational efficiency and classification performance [33]. A related two-stage detection-and-refinement strategy achieved leading performance in the MIDOG21 and MIDOG22 benchmarks, illustrating the continued benefit of separating candidate generation from final mitosis classification [34].

The Mitosis Domain Generalization (MIDOG) challenge series has become an important benchmark for evaluating the robustness of automated mitosis detection under domain shift [17]. MIDOG datasets intentionally incorporate variability related to scanners, institutions, staining conditions, and tissue appearance, providing a more realistic assessment of generalizability than conventional single-center datasets [17]. Methods developed for these challenges have explored stain augmentation and normalization strategies [29], adversarial domain adaptation [32], style-transfer approaches [35], Fourier-based augmentation [36], and self-supervised representation learning [37]. Ensemble-based strategies have also been used to improve robustness across heterogeneous image domains [38].

More recent work has expanded automated mitosis analysis beyond single-tumor datasets. Pan-cancer approaches have investigated whether mitotic morphology can be recognized consistently across multiple tissue types [39]. Other studies have focused on distinguishing typical from atypical mitotic figures, reflecting increasing interest in the biological information contained in mitotic morphology itself [40]. These developments suggest that automated mitosis analysis is evolving from a narrow detection problem toward a broader computational characterization of proliferative phenotypes.

Despite this progress, much of the existing literature remains concentrated on breast cancer or mixed-organ benchmark datasets [14]. Dedicated automated mitosis studies in LUSC remain comparatively limited [39]. This is important because LUSC has distinct architectural, cytological, stromal, and inflammatory characteristics that may influence the transferability of models developed in other tumor types [21]. Large public resources such as TCGA-LUSC provide diagnostic whole-slide images together with extensive clinical, genomic, and transcriptomic information, creating an opportunity to study mitotic activity within a broader biological context [5]. However, TCGA-LUSC does not provide exhaustive mitotic annotations, making direct supervised training difficult [41].

Transfer learning offers a practical strategy for addressing this limitation. Features learned from well-annotated source-domain datasets can be adapted to target tumor types with fewer annotations, potentially reducing the annotation burden while preserving useful morphological representations [42]. In computational pathology, lower-level features such as nuclear contours, chromatin texture, and local cellular organization may remain partially transferable across tissue types and higher-level representations can then be fine-tuned to account for the specific morphology and staining characteristics of LUSC [43]. Cross-cancer transfer from a well-annotated mitosis dataset such as MIDOG to TCGA-LUSC is therefore a biologically and computationally motivated approach [18,43].

An additional direction in computational pathology is the integration of image-derived phenotypes with clinical and molecular information [44]. Rather than treating automated mitosis detection solely as an image-classification task, quantitative measures of mitotic activity can be investigated in relation to patient survival, genomic instability, immune composition, signaling pathways, and therapeutic vulnerability [45]. Increased proliferative activity has been linked to cell-cycle dysregulation and genomic instability across multiple malignancies [7]. It may also be associated with changes in immune-cell composition and tumor-microenvironment interactions [45]. Transcriptomic analyses can further identify biological pathways enriched in highly proliferative tumors [46].

Gene set enrichment analysis provides a systematic framework for identifying coordinated pathway-level transcriptional changes associated with quantitative tumor phenotypes [46]. Computational drug-response approaches can additionally generate hypotheses regarding potential therapeutic vulnerabilities from tumor transcriptomic profiles [47]. Similarly, transcription-factor activity inference methods enable investigation of regulatory programs that may underlie highly proliferative tumor states [48]. Integrating these analyses with computationally quantified mitotic activity may therefore provide a more comprehensive view of LUSC biology.

Taken together, the literature indicates that deep-learning-based mitosis detection has achieved substantial technical progress, particularly through domain-generalization and transfer-learning strategies [17]. However, systematic application to LUSC and integration with clinical and multi-omics characteristics remain limited [21]. The present study addresses this gap by transferring a mitosis-classification model from MIDOG 2022 to TCGA-LUSC and deriving tumor-area mitosis proportion (TAMP) as a quantitative imaging measure of proliferative activity [49]. TAMP is subsequently evaluated in relation to overall survival, tumor-microenvironment characteristics, genomic features, pathway enrichment, predicted drug sensitivity, and inferred transcription-factor activity, extending automated mitosis analysis from image classification toward an integrated computational pathology framework [13].

## 3. Materials and Methods

### 3.1. Data Acquisition and Cohort Selection

This study used two complementary digital pathology cohorts to develop a transfer-learning framework for mitotic figure classification and to investigate the clinical and molecular correlates of image-derived mitotic activity in LUSC. The source domain comprised 150 hematoxylin-and-eosin (H&E)-stained human breast-carcinoma whole-slide images (WSIs) from the MIDOG dataset, acquired at 40× magnification with a nominal resolution of 0.25 µm/pixel. Expert mitotic-figure annotations provided the reference labels for source-domain model development. The MIDOG cohort was selected because it provides a controlled benchmark for mitosis recognition under scanner and staining variability [17,49].

TCGA-LUSC served as the target domain for model adaptation and downstream clinicomolecular analyses. Diagnostic WSIs and matched clinical and molecular data were obtained from The Cancer Genome Atlas LUSC project. Among 504 available cases, 478 patients contributing 512 diagnostic WSIs satisfied the predefined image-quality criteria and were retained for analysis. Slides with inadequate staining quality or insufficient evaluable tissue were excluded. The availability of matched histopathological, transcriptomic, genomic, and clinical information enabled the image-derived mitotic phenotype to be evaluated beyond classification performance and examined in relation to multiple dimensions of tumor biology.

### 3.2. Histopathological Annotation and Patch Generation

TCGA-LUSC diagnostic WSIs were independently reviewed using ASAP software (version 1.9) by two board-certified pathologists with expertise in thoracic pathology. Tumor regions of interest were manually delineated, and unequivocal mitotic figures within the tumor compartment were annotated according to conventional morphological criteria, including condensed basophilic chromosomes, loss of an intact nuclear membrane, and morphology consistent with active mitosis rather than apoptosis or necrosis. Interobserver agreement for mitotic assessment was quantified using Cohen’s κ, with κ ≥ 0.85 used as the prespecified agreement criterion. The observed interobserver agreement was κ = 0.87, indicating that the prespecified reliability criterion was satisfied. Discrepant annotations were resolved by joint consensus review, and the consensus annotations were used as reference labels.

Binary tumor masks were generated from the annotated regions. Non-overlapping RGB patches of 128 × 128 pixels were extracted at native 40× resolution. At 0.25 µm/pixel, each patch represented an approximately 32 µm × 32 µm tissue field, providing sufficient local nuclear context while maintaining computational efficiency. A patch was assigned a positive label when the centroid of at least one annotated mitotic figure fell within its spatial boundaries; all remaining tumor patches were labeled negative.

Background and tissue-poor patches were excluded using Otsu thresholding, which estimates an intensity threshold by maximizing between-class variance [50]. This preprocessing step reduced computational load and limited exposure of the classifier to visually uninformative regions.

### 3.3. ResNet-34 Architecture

Mitotic recognition was formulated as binary patch classification. The backbone was based on ResNet-34, whose residual formulation facilitates optimization of deep convolutional representations through identity-based skip connections [25].

For an input patch x, the network produced a scalar logit z(x), which was converted to a mitosis probability using the sigmoid function:

(1) $$\begin{array}{cccc} & P\left(y=1|x\right)=\sigma \left(z\right)=\frac{1}{1+e^{-z}}. & & \end{array}$$

The network contained an initial 7 × 7 convolution with 64 filters and a stride of 2, followed by 3 × 3 max pooling. Four residual stages were configured as 3, 4, 6, and 3 residual blocks with 64, 128, 256, and 512 channels, respectively. Each residual block consisted of two 3 × 3 convolutions combined with batch normalization and rectified linear unit activation. Global average pooling reduced the terminal feature representation to a 512-dimensional vector, followed by a single-neuron fully connected classification layer. ResNet-34 was selected to balance representational capacity and computational cost for local patch-level morphology classification.

### 3.4. Two-Stage Transfer Learning

A two-stage transfer-learning strategy was used to address morphological and staining differences between breast carcinoma and LUSC. In the first stage, the complete network was trained on MIDOG patches to learn transferable representations of mitotic morphology, including chromatin condensation, nuclear contour, texture, and local cellular context. In the second stage, the network was adapted to TCGA-LUSC. The initial convolutional layer, max-pooling layer, Stage 1, and Stage 2 were frozen, whereas Stages 3 and 4 remained trainable, allowing higher-level representations to adapt to LUSC-specific morphology while retaining lower-level visual features learned from MIDOG.

For the initial target-domain evaluation, TCGA-LUSC patches were stratified into 70% training, 15% validation, and 15% test partitions while preserving class proportions. This initial split was performed at the patch level and was used to quantify the effect of target-domain fine-tuning. Because patches from the same patient could occur in different partitions, patient overlap was subsequently assessed, and leakage-mitigated evaluation was performed using patient-level stratified five-fold cross-validation, as detailed in Section Data Leakage Assessment and Mitigation. In each fold, all patches from a given patient were assigned exclusively to either the training or held-out evaluation set. An additional WSI-level holdout analysis was performed as a complementary robustness assessment. Optimization was performed using the Adam optimizer with a batch size of 32 for a maximum of 30 epochs. Early stopping was applied when the validation loss failed to improve for five consecutive epochs. The same architecture, optimization settings, augmentation procedure, and fine-tuning strategy were retained across the patient-level cross-validation folds to ensure directly comparable performance estimates.

### 3.5. Class-Imbalance Handling and Data Augmentation

Mitotic patches represented approximately 3% of the available TCGA-LUSC patch population, creating substantial class imbalance. We therefore used Focal Loss, which reduces the contribution of easily classified examples and increases the relative influence of difficult minority-class observations [51]. For binary classification, Focal Loss was defined as

$$L_{FL}=-\alpha_{t}{\left(1-p_{t}\right)}^{\gamma }log\left(p_{t}\right),$$

where $p_{t}$ denotes the predicted probability assigned to the correct class, $\alpha_{t}$ controls class weighting, and γ determines the degree to which well-classified examples are down-weighted. In this study, α=0.25 and γ=2.0 were used. Training-only augmentation was implemented with Albumentations [52]. Geometric transformations included horizontal and vertical flips, 90° rotations, and affine transformations with rotation within ±15°, translation up to 10% of patch dimensions, and scaling between 0.90 and 1.10. Color variability was introduced in HSV space using hue shifts of ±18 and saturation/value shifts of ±20. Gaussian noise with variance sampled from 5.0 to 30.0 and Gaussian blur with kernel sizes of 3 × 3 or 5 × 5 and σ between 0.1 and 2.0 were applied with probability 0.3.

All images were normalized using ImageNet channel statistics. Validation and test patches underwent normalization only and were not augmented.

### 3.6. Performance Evaluation and Model Interpretation

Model performance was evaluated using the area under the receiver operating characteristic curve (AUC), area under the precision–recall curve (PR-AUC), sensitivity, specificity, precision, accuracy, recall, and F1-score. PR-AUC was included as an imbalance-sensitive metric because mitosis-positive patches represented approximately 3% of the TCGA-LUSC patch population. For a classification threshold t, the F1-score was calculated as

$$F1=2\frac{Precision\times Recall}{Precision+Recall}.$$

The final operating threshold was determined from the validation set by maximizing the Youden index,

J(t)=Sensitivity(t)+Specificity(t)−1.

Source-domain performance was evaluated on the held-out MIDOG test set. Following target-domain fine-tuning, performance was initially assessed on the TCGA-LUSC patch-level validation and test partitions. Because patch-level partitioning can place patches from the same patient in different subsets, additional patient- and slide-level validation procedures were performed as described in Section Data Leakage Assessment and Mitigation.

Model interpretability was examined using Gradient-weighted Class Activation Mapping (Grad-CAM) [53]. Gradients associated with the mitosis-positive output were propagated to the final convolutional layer to identify image regions contributing most strongly to each prediction. The resulting activation maps were overlaid on the corresponding H&E patches and qualitatively compared with pathologist-annotated mitotic structures.

#### Data Leakage Assessment and Mitigation

Potential information leakage arising from the initial patch-level partitioning of TCGA-LUSC was assessed by tracing each extracted patch to its originating patient. Among 478 patients, 142 (29.7%) contributed patches to more than one data partition, including 67 patients (14.0%) represented in both the training and test sets. This overlap indicated that the original patch-level evaluation could provide an optimistically biased estimate of generalization performance.

To obtain a patient-independent performance estimate, we therefore implemented stratified five-fold cross-validation at the patient level. All patches originating from a given patient were assigned exclusively to one fold, preventing the same patient from contributing data to both model training and held-out evaluation within any cross-validation iteration. The model was re-trained separately for each fold using the architecture, optimization parameters, augmentation procedures, and fine-tuning strategy specified in Section 3.3, Section 3.4 and Section 3.5. Within each fold, model selection, early stopping, and operating-threshold determination were performed using a patient-disjoint validation subset drawn exclusively from the training patients. The held-out patients were used only for final fold evaluation.

The fold-level AUCs were 0.86, 0.87, 0.88, 0.89, and 0.90, yielding a mean AUC of 0.88. The reported 95% confidence interval was calculated from these five-fold-level AUC estimates using a one-sample t-based interval,

$$\bar{AUC}\pm t_{0.975,4}\frac{SD}{\sqrt{5}},$$

which yielded a 95% CI of 0.860–0.900, reported as 0.86–0.90. No bootstrap resampling was used to derive this primary confidence interval. Because the interval was estimated from only five-fold-level observations, it should be interpreted as a measure of cross-fold variability with limited degrees of freedom rather than as a patient-level bootstrap estimate of uncertainty.

Patient-level cross-validation therefore yielded a mean AUC of 0.88 (95% CI, 0.86–0.90), compared with an AUC of 0.93 under the original patch-level partitioning. The absolute decrease was 0.05, corresponding to an approximately 5.4% relative reduction. Nevertheless, the leakage-mitigated AUC remained higher than the AUC of 0.81 obtained when the MIDOG-trained model was applied directly to TCGA-LUSC before target-domain fine-tuning, supporting a target-domain benefit from LUSC-specific adaptation.

As an additional robustness assessment, a patient-disjoint holdout cohort was constructed by assigning patients, rather than individual WSIs, to either the training or holdout set. All WSIs from a given patient were assigned exclusively to one set. The holdout cohort comprised 95 patients (19.9% of the cohort) contributing 102 WSIs (19.9% of all WSIs), all of which were excluded from model training. This analysis yielded an AUC of 0.87 (95% CI, 0.84–0.90), closely agreeing with the patient-level cross-validation estimate.

For all downstream analyses, TAMP was calculated from patient-level out-of-fold predictions. Each patient’s TAMP therefore originated from a fold-specific model that had not been trained on patches from that patient. Survival, genomic, transcriptomic, immune, regulatory, and predicted drug-response analyses were consequently performed using these out-of-fold TAMP estimates rather than predictions generated under the original patch-level partitioning.

### 3.7. Tumor-Area Mitosis Proportion

The fine-tuned network was used to derive a quantitative imaging measure of mitotic activity across TCGA-LUSC. For all downstream analyses, predictions were generated using the patient-level five-fold cross-validation framework described in Section Data Leakage Assessment and Mitigation. Each patient therefore received out-of-fold predictions from a model that had not been trained on patches from that patient. Tissue-containing tumor patches were assigned a mitosis probability, and patches exceeding the validation-derived Youden threshold were classified as mitosis-positive. For slide s, TAMP was defined as

$${TAMP}_{s}=\frac{N_{s}^{+}}{N_{s}^{tumor}},$$

where $N_{s}^{+}$ denotes the number of tumor-region patches classified as mitosis-positive and $N_{s}^{tumor}$ denotes the total number of evaluable patches within the pathologist-defined tumor mask.

A complementary whole-slide mitosis proportion was calculated using all evaluable tissue patches as the denominator. TAMP was selected as the primary downstream imaging phenotype because restriction to the tumor compartment reduces dilution by adjacent normal and other non-neoplastic tissue. TAMP represents the proportion of model-positive tumor patches and is therefore distinct from a conventional manually enumerated mitotic count per high-power field. For patients contributing more than one diagnostic WSI, slide-level measurements were aggregated to obtain a patient-level TAMP value. Patients were subsequently classified into high- and low-TAMP groups using the cohort median for survival and molecular comparisons. All survival, genomic, transcriptomic, immune, regulatory, and predicted drug-response analyses were performed using these patient-level out-of-fold TAMP estimates rather than predictions generated from the original patch-level partitioning.

### 3.8. Tumor Microenvironment and Genomic Feature Analysis

Patient-level out-of-fold TAMP estimates were matched with available tumor-microenvironment and genomic characteristics in the TCGA-LUSC cohort. Tumor-microenvironment variables included stromal fraction, leukocyte fraction, and selected immune-cell fractions. Genomic variables included indel-derived and SNV-derived neoantigen loads, indel burden, mutation-rate measures, and chromosomal alteration characteristics. Continuous associations between TAMP and these variables were evaluated using Spearman’s rank correlation. Analyses were restricted to patients with matched data available for the corresponding molecular variable. Because the tumor-microenvironment and genomic analyses were exploratory, these comparisons were not treated as confirmatory hypothesis tests. Nominal, unadjusted p-values are reported for descriptive purposes and are explicitly distinguished from FDR-corrected results. No claim of statistical significance after multiple-testing correction is made for these exploratory associations.

### 3.9. Transcriptomic and Pathway Analysis

RNA-sequencing data matched to the TCGA-LUSC imaging cohort were analyzed to characterize transcriptional differences associated with TAMP. Differential expression between high- and low-TAMP tumors was performed using DESeq2 [54], which models RNA-seq count data using negative-binomial generalized linear models. Gene Set Enrichment Analysis was conducted using clusterProfiler against Gene Ontology, KEGG, and Hallmark collections from MSigDB. Genes were ranked according to the differential-expression statistic, and enrichment was evaluated across the ordered gene list. Pathways with a Benjamini–Hochberg false-discovery rate below 0.05 were considered significant.

This pathway-level analysis was intended to determine whether the image-derived proliferative phenotype was associated with coordinated biological programs rather than isolated expression changes.

### 3.10. Predicted Drug Response

Transcriptome-based drug response was estimated using oncoPredict. The method transfers drug-response relationships learned from pharmacogenomic reference datasets to tumor expression profiles and generates predicted half-maximal inhibitory concentration values for individual compounds.

For drug d and patient i, the inferred response was represented as

(2) $$\begin{array}{cccc} & {\hat{IC}}_{50}^{\left(i,d\right)}=f_{d}\left(x_{i}\right), & & \end{array}$$

where $x_{i}$ represents the patient-specific transcriptomic profile and $f_{d}$ denotes the drug-specific predictive model. Predicted IC_50_ distributions were compared between high- and low-TAMP tumors. These estimates were treated as computational hypotheses of therapeutic susceptibility rather than experimental measurements of drug efficacy.

### 3.11. Transcription-Factor Activity Inference

Regulatory activity was investigated using pySCENIC and DoRothEA [55]. SCENIC reconstructs transcriptional regulatory programs from transcription-factor–target relationships and gene-expression patterns, whereas DoRothEA provides curated regulons for estimating transcription-factor activity from target-gene expression.

For each transcription factor k, an activity score $A_{ik}$ was inferred for patient i from the coordinated expression of its regulon. Activity distributions were then compared between the high- and low-TAMP groups to identify regulatory programs associated with increased mitotic activity.

TF–target interaction networks were visualized using Cytoscape to support interpretation of candidate upstream regulatory programs.

### 3.12. Statistical and Survival Analysis

All statistical analyses were performed in R version 4.3.0. Continuous associations between TAMP and clinicopathological or molecular variables were evaluated using Spearman’s rank correlation coefficient. Two-group comparisons were performed using the Mann–Whitney U test, whereas variables with three or more categories were assessed using the Kruskal–Wallis test.

Overall survival was defined as the primary clinical endpoint. Patients were stratified according to median TAMP, and survival functions were estimated using the Kaplan–Meier estimator. Group differences were assessed with the log-rank test.

The prognostic association of TAMP was initially quantified using univariable Cox proportional-hazards regression:

$$h\left(t|X\right)=h_{0}\left(t\right)exp\left(\beta X\right),$$

where h(t∣X) is the hazard at time t, $h_{0}\left(t\right)$ is the baseline hazard, and X denotes TAMP group membership. The corresponding hazard ratio was calculated as

$$HR=e^{\beta }.$$

To determine whether the association between TAMP and overall survival was independent of established clinicopathological factors, a multivariable Cox proportional-hazards model was subsequently fitted with TAMP group, age at diagnosis, sex, and pathological stage as covariates. Age was modeled as a continuous variable, whereas TAMP group, sex, and pathological stage were entered as categorical variables. Low TAMP, female sex, and Stage 1 disease served as the reference categories. Patients with missing data for any included variable were excluded using complete-case analysis. Adjusted hazard ratios, 95% confidence intervals, and two-sided p-values were reported.

The proportional-hazards assumption was assessed using Schoenfeld residuals. No statistically significant violation was detected at the 0.05 level. For confirmatory analyses involving multiple simultaneous hypotheses, including differential expression, pathway enrichment, and drug-response comparisons, Benjamini–Hochberg correction was used to control the false-discovery rate at 5%. Tumor-microenvironment and genomic correlation analyses were treated as exploratory; their p-values were reported as nominal and unadjusted and were not interpreted as evidence of statistical significance after multiple-testing correction.

### 3.13. Computational Environment

Model development and inference were performed under Ubuntu 22.04 on an NVIDIA A100 GPU with 40 GB memory and 128 GB system RAM. The deep-learning pipeline was implemented in Python 3.9.18 using PyTorch 2.0.1 and CUDA 11.8. Statistical and molecular analyses were performed in R 4.3.0. The principal software components included Albumentations 1.3.1, DESeq2 1.38.3, clusterProfiler 4.6.0, pySCENIC 0.12.1, DoRothEA 1.9.0, Cytoscape 3.10.0, and the survival package 3.5–5.

## 4. Results

### 4.1. Model Performance and Leakage-Mitigated Validation

Source-domain model development used patches extracted from 150 annotated MIDOG breast-carcinoma cases. Because non-mitotic tissue patches substantially outnumbered mitosis-positive patches, 50,000 non-mitotic patches were randomly sampled as training controls. Source-domain performance was evaluated on a separate held-out MIDOG test set containing 2000 patches, including 821 mitosis-positive and 1179 non-mitotic patches. For target-domain adaptation, 478 TCGA-LUSC patients contributing 512 diagnostic WSIs were retained after quality control. Interobserver agreement for mitotic annotation was high (κ=0.87) and exceeded the prespecified agreement criterion of κ≥0.85. Cohort composition and evaluation strategies are summarized in Table 1.

On the internal MIDOG test set, the model achieved an AUC of 0.972 (95% CI, 0.965–0.980). At the selected operating threshold, the confusion matrix contained 811 true positives, 10 false negatives, 189 false positives, and 990 true negatives, corresponding to sensitivity 0.988, specificity 0.840, precision 0.811, F1-score 0.891, and accuracy 0.901 (Figure 1). The error profile was characterized by a low false-negative rate, with most errors arising from non-mitotic patches classified as mitotic. When transferred to TCGA-LUSC without target-domain fine-tuning, the model achieved an AUC of 0.81. Fine-tuning produced an initial patch-level AUC of 0.93. Subsequent patient tracing, however, identified overlap across the original patch partitions: 142 of 478 patients (29.7%) contributed patches to more than one partition, including 67 patients (14.0%) represented in both the training and test sets (Figure 1). The initial patch-level result was therefore interpreted as potentially optimistic, and patient-level stratified five-fold cross-validation was performed to obtain a patient-independent estimate of performance.

Patient-level stratified five-fold cross-validation yielded a mean AUC of 0.88 (95% CI, 0.86–0.90), compared with an AUC of 0.93 under the initial patch-level partition. The absolute AUC reduction was 0.05, corresponding to an approximately 5.4% relative reduction. Importantly, patient-level performance remained above the pre-fine-tuning TCGA-LUSC AUC of 0.81, supporting a target-domain benefit from LUSC-specific adaptation. Given the approximately 3% prevalence of mitosis-positive patches in TCGA-LUSC, additional imbalance-sensitive metrics were evaluated. Patient-level five-fold cross-validation yielded a mean PR-AUC of 0.24, with a precision of 0.11, a recall of 0.72, and an F1-score of 0.19 at the validation-derived operating threshold. A complementary WSI-level holdout analysis reserving 20% of WSIs (n=102) yielded an AUC of 0.87 (95% CI, 0.84–0.90), a PR-AUC of 0.22, a precision of 0.10, a recall of 0.70, and an F1-score of 0.18. The close agreement in ROC-AUC between the patient-level cross-validation and WSI-level holdout analyses supports substantial target-domain discrimination, while the lower precision and PR-AUC values reflect the marked class imbalance in the target dataset. All downstream TAMP analyses used patient-level out-of-fold predictions rather than predictions generated under the original patch-level partition. Grad-CAM activation was concentrated around morphologically plausible mitotic structures, providing qualitative evidence that predictions relied on relevant nuclear regions.

### 4.2. Ablation Analysis of Transfer-Learning Components

To evaluate the contribution of individual components of the proposed transfer-learning framework, we performed ablation experiments using the same patient-level stratified five-fold cross-validation strategy applied in the primary target-domain evaluation. All ablation models were trained and evaluated using identical patient-level folds, optimization settings, model-selection criteria, and threshold determination procedures. Only the component under evaluation was modified.

The complete pipeline, consisting of MIDOG pre-training, selective layer freezing during target-domain adaptation, Focal Loss optimization, and the full augmentation strategy, achieved a mean AUC of 0.88 (95% CI, 0.86–0.90) and a mean PR-AUC of 0.24. Removing MIDOG pre-training and training the model from random initialization resulted in the largest performance reduction, decreasing the mean AUC to 0.79 (95% CI, 0.76–0.82) and the mean PR-AUC to 0.11. This finding indicates that source-domain knowledge transfer provided the largest contribution to target-domain generalization.

Removing selective layer freezing by allowing full-network fine-tuning resulted in a smaller reduction in performance, with a mean AUC of 0.85 (95% CI, 0.82–0.87) and a mean PR-AUC of 0.19. Replacing Focal Loss with weighted binary cross-entropy produced a mean AUC of 0.86 (95% CI, 0.83–0.88) and a mean PR-AUC of 0.20 (Table 2). Removing the training augmentation strategy yielded a mean AUC of 0.86 (95% CI, 0.84–0.89) and a mean PR-AUC of 0.21.

Overall, the ablation analysis demonstrated that MIDOG pre-training was the most influential component of the framework, while selective layer freezing, loss formulation, and augmentation provided additional incremental improvements. These results support the use of transfer learning as the primary mechanism for adapting mitotic morphology representations from the source domain to LUSC whole-slide images.

### 4.3. Survival Analysis

The association between patient-level out-of-fold TAMP and OS was evaluated after stratification at the cohort median. High-TAMP patients showed significantly poorer OS than low-TAMP patients (log-rank p=0.017; Figure 2). Univariable Cox proportional-hazards regression yielded an HR of 1.73 (95% CI, 1.12–2.68; p=0.014), corresponding to a 73% higher hazard of death in the high-TAMP group (Table 3). In the multivariable Cox model, high TAMP remained significantly associated with poorer OS after adjustment for age, sex, and pathological stage (adjusted HR = 1.62, 95% CI, 1.03–2.54; p=0.036; (Table 3)). Age was independently associated with OS (adjusted HR per year = 1.02, 95% CI, 1.01–1.03; p=0.006). Compared with Stage 1 disease, Stage 3 (adjusted HR = 2.02, 95% CI, 1.28–3.18; p=0.003) and Stage 4 disease (adjusted HR = 2.60, 95% CI, 1.39–4.86; p=0.003) were associated with poorer OS. Sex and Stage 2 disease were not significantly associated with OS in the adjusted model.

Because TAMP values were generated from out-of-fold predictions, the survival analysis did not use predictions from models trained on the corresponding patients. These findings indicate that TAMP retained prognostic information after adjustment for the included clinicopathological variables within TCGA-LUSC. However, its independent prognostic value and incremental performance beyond established clinical models require confirmation in external cohorts.

### 4.4. Tumor Microenvironment Analysis

The association between patient-level out-of-fold TAMP estimates and the tumor microenvironment was examined across stromal and immune compartments (Figure 3). In these exploratory analyses, TAMP showed a weak inverse association with stromal fraction (ρ=−0.117, nominal unadjusted p=0.041), whereas overall leukocyte fraction was unrelated to TAMP (ρ=−0.012, nominal unadjusted p=0.833). At the immune-cell subtype level, increasing TAMP showed a nominal negative association with resting memory CD4^+^ T-cell fraction (ρ=−0.158, unadjusted p=0.010) and nominal positive associations with activated mast-cell fraction (ρ=0.129, unadjusted p=0.034) and M1 macrophage fraction (ρ=0.105, unadjusted p=0.025) (Figure 3). These p-values were not corrected for multiple testing and should therefore be interpreted as exploratory associations rather than statistically validated findings. The small effect sizes indicate selective and modest microenvironmental differences rather than broad immune enrichment.

### 4.5. Genomic Feature Analysis

The association between patient-level out-of-fold TAMP estimates and genomic mutation characteristics was examined to determine whether increased mitotic activity was related to features of genomic alteration. In these exploratory analyses, TAMP showed a weak positive association with indel-derived neoantigen load (ρ=0.147, nominal unadjusted p=0.013; Figure 4). Because these genomic analyses were exploratory, the reported p-values were not corrected for multiple testing and should not be interpreted as statistically validated findings. Additional genomic features, including SNV-derived neoantigen load, silent and nonsilent mutation rates, and measures of chromosomal segmentation and fragmentation, showed generally small associations with TAMP. Overall, these findings do not support TAMP as a surrogate for generalized genomic instability but suggest that it may capture limited information associated with selected genomic characteristics.

### 4.6. Pathway Enrichment Analysis and Molecular Mechanisms

Gene set enrichment analysis identified coordinated transcriptional programs associated with patient-level out-of-fold TAMP. Patients were divided into high- and low-TAMP groups according to the cohort median, and genes were ranked using differential-expression statistics. Positive enrichment scores therefore indicate preferential enrichment toward the high-TAMP end of the ranked gene list. The GOBP_REGULATION_OF_STEM_CELL_PROLIFERATION gene set was enriched in high-TAMP tumors (ES = 0.555, FDR < 0.001; Figure 5A). Leading-edge genes included regulators associated with proliferative and stemness-related processes, including MYC and components of WNT/β-catenin signaling, whereas differentiation-associated genes, including members of the BMP family, were preferentially represented toward the low-TAMP end of the ranked profile. The GOBP_REGULATION_OF_CELLULAR_RESPONSE_TO_GROWTH_FACTOR_STIMULUS gene set was also enriched in the high-TAMP group (ES = 0.528, FDR = 0.002; Figure 5B), with leading-edge genes linked to MAPK and PI3K–AKT signaling. These findings provide transcriptomic context for the image-derived high-TAMP phenotype but represent pathway-level associations rather than evidence of causal pathway activation. GSEA does not directly measure functional pathway activation or establish causality; accordingly, the enrichment of genes related to WNT/β-catenin, MAPK, and PI3K–AKT signaling indicates their association with the high-TAMP transcriptional profile but does not demonstrate that elevated mitotic activity directly activates these pathways.

### 4.7. Predicted Drug-Response Results

Transcriptome-derived predicted drug-response values were compared between high- and low-TAMP tumors using patient-level out-of-fold TAMP estimates. High-TAMP tumors showed lower predicted IC_50_ values for palbociclib, alisertib, and AZD7762 than low-TAMP tumors (all *p* < 0.001; Figure 6). These compounds target distinct components of cell-cycle regulation, including CDK4/6, Aurora kinases, and checkpoint kinases, respectively. In contrast, no significant difference was observed for erlotinib (*p* = 0.521). The pattern was therefore selective rather than uniform across evaluated compounds and was more pronounced for agents targeting cell-cycle-associated processes. These transcriptome-derived predictions identify hypothesis-generating differences in computational drug-response estimates rather than experimentally measured treatment sensitivity.

### 4.8. Transcription-Factor Activity Results

Inferred transcription-factor activity was also associated with TAMP status. MYC, E2F1, FOXM1, and TCF7L2 showed higher inferred activity in high-TAMP tumors, whereas SMAD4 and KLF4 showed lower inferred activity (all *p* < 0.001). These regulatory estimates are computational inferences from transcriptomic data and are interpreted as molecular correlates of the TAMP phenotype rather than direct biochemical measurements. The increased inferred activity of MYC, E2F1, and FOXM1 is consistent with the highly proliferative phenotype identified by histological mitotic activity and complements the pathway-level enrichment observed in Section 4.5. Conversely, the reduced inferred activity of SMAD4 and KLF4 indicates additional differences in regulatory programs associated with differentiation and growth control.

Together, these analyses provide hypothesis-generating evidence linking elevated image-derived mitotic activity with proliferation-associated regulatory programs and predicted sensitivity to selected cell-cycle-targeting compounds. However, both TF activities and drug responses were computationally inferred from transcriptomic data. Accordingly, the observed differences should be regarded as hypothesis-generating associations and require experimental validation before therapeutic implications can be established.

## 5. Discussion

We developed a transfer-learning framework that links automated mitosis recognition to an interpretable patient-level imaging phenotype in LUSC. The principal target-domain finding was the reduction in AUC from 0.93 under the initial patch-level partition to 0.88 under patient-level cross-validation. Patient tracing showed that the original partitions contained overlapping patients, which produced an optimistic estimate of target-domain performance. The patient-level AUC nevertheless remained higher than the pre-fine-tuning AUC of 0.81, indicating that LUSC-specific adaptation improved discrimination beyond direct application of the source-domain model. These results demonstrate the importance of patient-level separation when evaluating computational pathology models and are consistent with their documented sensitivity to tissue morphology, staining variation, and image-acquisition conditions [17,28,29].

The model error profile provides additional context for interpreting TAMP. On the MIDOG test set, the classifier missed 10 mitotic patches and incorrectly classified 189 non-mitotic patches as positive. The selected operating point therefore favored sensitivity over specificity. This behavior reduces the number of missed mitotic events but may increase positive predictions when the model is applied across extensive tumor regions. This issue is particularly relevant because the prevalence of mitosis-positive patches was substantially higher in the MIDOG test set than in TCGA-LUSC. Precision and F1-score are prevalence-dependent and may consequently overestimate positive predictive performance in the target domain. TAMP should therefore be interpreted as a model-derived imaging phenotype rather than a direct replacement for conventional mitotic counting.

We defined TAMP as the proportion of evaluable tumor-region patches classified as mitosis-positive. All downstream analyses used patient-level out-of-fold predictions, ensuring that each patient’s TAMP was generated by a model that had not been trained on patches from that patient. This design reduced the possibility that the observed clinical and molecular associations resulted from training and evaluation overlap. Grad-CAM activation was concentrated around morphologically plausible mitotic structures, providing qualitative evidence that the classifier used relevant nuclear regions. However, Grad-CAM does not establish prediction validity and cannot replace quantitative localization or external validation.

High TAMP was associated with poorer OS. The Kaplan–Meier analysis showed a significant difference between the high- and low-TAMP groups (log-rank p=0.017). In the univariable Cox model, high TAMP was associated with a 73% higher hazard of death (HR = 1.73, 95% CI, 1.12–2.68; p=0.014). After adjustment for age, sex, and pathological stage, high TAMP remained significantly associated with poorer OS (adjusted HR = 1.62, 95% CI, 1.03–2.54; p=0.036). The modest attenuation after adjustment indicates that the association was not explained entirely by the included clinicopathological variables. Age and advanced pathological stage were also independently associated with poorer survival, whereas sex and Stage 2 disease were not significant in the adjusted model.

The direction of the survival association is biologically plausible because increased cellular proliferation is associated with aggressive tumor behavior, and mitotic assessment contributes to grading or classification systems in other malignancies [10,11]. Our approach extends this principle by quantifying mitotic activity across pathologist-defined tumor regions using a reproducible computational procedure. However, the adjusted analysis does not establish clinical utility. The model included only the clinicopathological variables available in TCGA-LUSC and may remain affected by residual confounding from treatment, performance status, comorbidities, smoking exposure, and other prognostic factors. In addition, the median TAMP threshold was derived and evaluated within the same cohort. External validation with a prespecified threshold and formal assessment of incremental prognostic performance are required to determine whether TAMP adds clinically relevant information to established prognostic models.

The immune and genomic associations were weaker than the survival and transcriptomic findings. TAMP showed a weak inverse correlation with stromal fraction (ρ=−0.117, p=0.041), whereas leukocyte fraction was unrelated to TAMP (ρ=−0.012, p=0.833). Resting memory CD4^+^ T-cell fraction was negatively correlated with TAMP (ρ=−0.158, p=0.010), while activated mast-cell fraction (ρ=0.129, p=0.034) and M1 macrophage fraction (ρ=0.105, p=0.025) showed weak positive correlations. These small effect sizes do not support broad immune enrichment in highly mitotic tumors. Instead, they indicate modest differences in selected stromal and immune-cell populations. Previous computational pathology studies showed that histological organization can reflect immune and molecular characteristics [41,45]. Our findings extend this relationship to a proliferation-related imaging phenotype in LUSC, but the nominal associations should be interpreted as exploratory because multiple immune variables were evaluated.

Genomic associations were similarly modest. TAMP showed a weak positive correlation with indel-derived neoantigen load (ρ=0.147, nominal unadjusted p=0.013), whereas its association with overall indel burden did not reach nominal statistical significance (ρ=0.110, unadjusted p=0.056). Other mutation-related variables showed small associations. These findings do not support TAMP as a surrogate for generalized genomic instability. Instead, TAMP appears to capture a limited component of selected genomic characteristics. This interpretation is consistent with evidence that histological patterns in NSCLC contain probabilistic information about underlying molecular alterations [41]. The small effect sizes and multiple exploratory comparisons require conservative interpretation. The strongest molecular associations occurred at the transcriptomic and regulatory levels. Gene set enrichment analysis identified enrichment of GOBP_REGULATION_OF_STEM_CELL_PROLIFERATION in high-TAMP tumors (ES = 0.555, FDR < 0.001), with contributions from MYC and genes associated with WNT/β-catenin signaling.

GOBP_REGULATION_OF_CELLULAR_RESPONSE_TO_GROWTH_FACTOR_STIMULUS was also enriched (ES = 0.528, FDR = 0.002), with genes related to MAPK and PI3K-AKT signaling represented in the enriched profile. These results indicate that high TAMP is associated with coordinated transcriptional programs related to proliferative capacity, stem-cell regulation, and growth-factor responses. The opposing representation of differentiation-associated genes, including BMP-family members, further indicates distinct transcriptional states between the TAMP groups. However, enrichment analysis identifies coordinated expression patterns and does not demonstrate causal or functional activation of individual signaling pathways.

The inferred transcription-factor profiles supported the transcriptomic findings. MYC, E2F1, FOXM1, and TCF7L2 showed higher inferred activity in high-TAMP tumors, whereas SMAD4 and KLF4 showed lower activity. Increased inferred activity of regulators associated with cell-cycle progression and proliferation was concordant with both the histological phenotype and the gene set enrichment results. This agreement across image-derived morphology, gene-expression programs, and inferred regulatory activity suggests that high TAMP reflects a broader proliferative molecular state. However, transcription-factor activity was inferred from bulk transcriptomic data and should not be interpreted as a direct biochemical measurement of regulatory activation.

Predicted drug-response analysis provided additional hypothesis-generating findings. High-TAMP tumors showed lower predicted IC_50_ values for palbociclib, alisertib, and AZD7762 (all p<0.001), whereas erlotinib showed no significant difference (p=0.521). The drugs associated with lower predicted IC_50_ values primarily target cell-cycle-related mechanisms, which is consistent with the increased mitotic activity and transcriptomic findings. However, oncoPredict generates transcriptome-derived estimates rather than measured patient responses [47]. These results therefore do not establish treatment sensitivity or support clinical treatment selection. Experimental validation and analysis of clinically treated cohorts are required.

The principal contribution of this study lies in connecting an interpretable histomorphological process with patient-level survival and molecular characteristics in LUSC. Previous studies established the technical feasibility of deep-learning-based mitosis detection [14,15,17,36,37,38,39,40] and showed that WSI-derived representations can encode clinically and molecularly relevant information [41,42,43,44]. We extend this work by deriving a patient-level measure of mitotic activity from leakage-mitigated out-of-fold predictions and examining its clinical and molecular associations. The transcriptomic and regulatory results provided the strongest molecular evidence, whereas the immune and genomic associations were modest and exploratory.

Overall, TAMP was associated with poorer OS and selected molecular characteristics in TCGA-LUSC. Patient-level cross-validation showed that substantial target-domain discrimination remained after addressing overlap in the initial patch-level partitions. Multivariable analysis further showed that TAMP retained prognostic information after adjustment for age, sex, and pathological stage. These findings support further investigation of TAMP as a research-derived proliferative imaging phenotype. Independent multicenter validation, standardized threshold selection, prospective clinical assessment, and experimental confirmation of the molecular and pharmacological findings are required before clinical application.

## 6. Limitations

This study has several limitations. First, the analysis was retrospective and based on a single public cohort, TCGA-LUSC, without independent multicenter external validation. Second, the initial patch-level split contained substantial patient overlap. Although the principal analyses were subsequently based on patient-level out-of-fold predictions from leakage-mitigated cross-validation, model evaluation remains internal to TCGA-LUSC. Third, the 128 × 128-pixel patch design captures local nuclear morphology but provides limited information about broader tissue architecture, spatial organization, and intratumoral heterogeneity.

Fourth, the ablation analysis evaluated the principal components of the transfer-learning framework using one-component-at-a-time modifications under a common patient-level cross-validation design. Although this approach provides evidence regarding the contribution of MIDOG pre-training, selective layer freezing, loss formulation, and data augmentation, it does not exhaustively evaluate interactions among these components or the individual effects of specific augmentation operations. Fifth, although high TAMP remained significantly associated with poorer overall survival after adjustment for age, sex, and pathological stage, the multivariable analysis was restricted to the clinicopathological variables available in TCGA-LUSC. Residual confounding by treatment, performance status, comorbidities, smoking exposure, and other prognostic factors cannot be excluded. Moreover, the median TAMP threshold was derived from the same cohort in which its prognostic association was evaluated. External validation using a prespecified threshold is therefore required.

Sixth, the MIDOG test set had a substantially higher proportion of mitosis-positive patches than TCGA-LUSC. Because precision, PR-AUC, and F1-score depend on class prevalence, performance estimates obtained from MIDOG may not represent positive-class performance in the target clinical domain. Seventh, the immune, genomic, transcriptomic, and pathway-level findings are observational and do not establish causal relationships between mitotic activity and the corresponding molecular characteristics. The immune and genomic analyses involved multiple exploratory comparisons; therefore, nominally significant associations should be interpreted cautiously unless they remain significant after false-discovery-rate correction. Eighth, transcription-factor activities were computationally inferred from bulk transcriptomic profiles and do not constitute direct biochemical measurements of regulatory activity. Ninth, drug-response estimates were generated using oncoPredict and do not represent experimentally measured or clinically observed treatment responses.

Tenth, TAMP is a model-derived imaging phenotype defined as the proportion of tumor-region patches classified as mitosis-positive. It is distinct from a conventional manually enumerated mitotic count per high-power field and may be affected by false-positive predictions, tumor-region delineation, tissue quality, and the selected classification threshold.

Before clinical translation, the method requires prospective multicenter validation across different scanners, staining protocols, and patient populations. The TAMP threshold, image-quality requirements, tumor-region selection, and reporting procedure must be standardized. Clinical implementation would also require integration into digital pathology workflows, pathologist oversight, and evidence that TAMP improves prognostic assessment beyond established clinicopathological factors.

## 7. Conclusions

We developed and internally evaluated a transfer-learning framework for automated quantification of mitotic activity in LUSC. The source-domain model achieved an AUC of 0.972 on the internal MIDOG test set. After adaptation to TCGA-LUSC, target-domain fine-tuning improved the initial patch-level AUC from 0.81 to 0.93. However, patient tracing revealed overlap among the original patch-level partitions, indicating that this estimate may have been optimistically biased. Under leakage-mitigated patient-level stratified five-fold cross-validation, the model achieved a mean AUC of 0.88 (95% CI, 0.86–0.90), while the complementary patient-disjoint WSI holdout evaluation yielded an AUC of 0.87 (95% CI, 0.84–0.90). These findings highlight the importance of patient-level separation for reliable assessment of computational pathology model generalizability.

Using patient-level out-of-fold predictions, we found that high TAMP was associated with poorer overall survival in univariable analysis (HR = 1.73, 95% CI, 1.12–2.68). This association remained significant after adjustment for age, sex, and pathological stage (adjusted HR = 1.62, 95% CI, 1.03–2.54; p=0.036). Transcriptomic and regulatory analyses linked high TAMP with proliferation- and growth-related programs, whereas immune, genomic, and predicted drug-response analyses revealed modest and hypothesis-generating associations.

TAMP represents an interpretable image-derived phenotype that links mitotic morphology with clinical outcome and molecular characteristics in TCGA-LUSC. However, it remains a research-derived measure rather than a clinically validated biomarker. Prospective multicenter validation, patient-disjoint external evaluation, standardized threshold definition, assessment of incremental clinical value, and experimental confirmation of molecular and pharmacological associations are required before any consideration of clinical implementation.

## Figures and Tables

**Figure 1:**
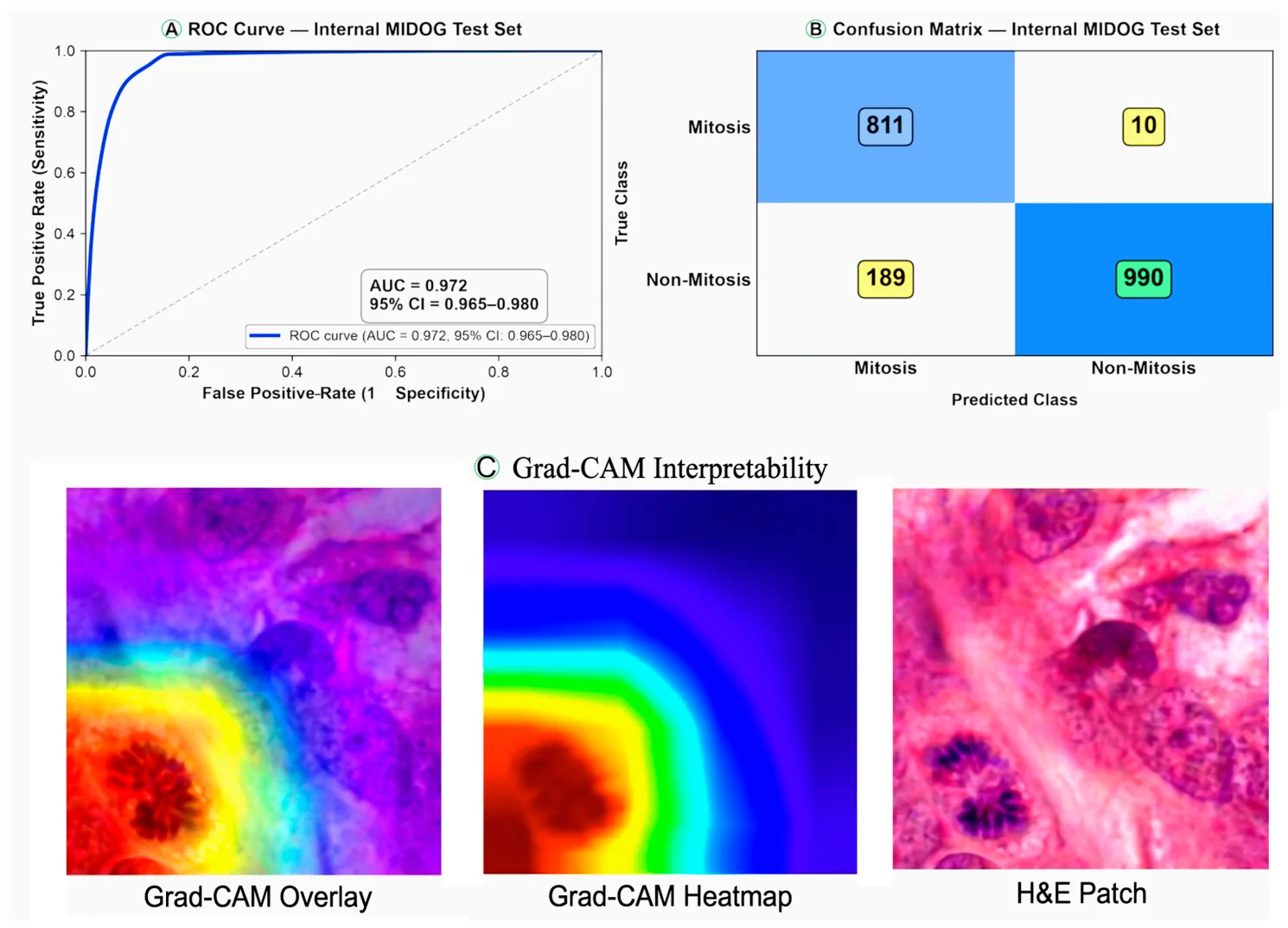
Model performance and interpretability on the internal MIDOG test set (*n* = 2000 patches). (**A**) Receiver operating characteristic curve for patch-level mitosis classification, yielding an AUC of 0.972 (95% CI: 0.965–0.980). (**B**) Confusion matrix at the Youden-optimal operating threshold, showing 811 true positives, 10 false negatives, 189 false positives, and 990 true negatives. (**C**) Grad-CAM overlay, heatmap, and corresponding H&E patch illustrating model attention around a representative mitotic figure.

**Figure 2:**
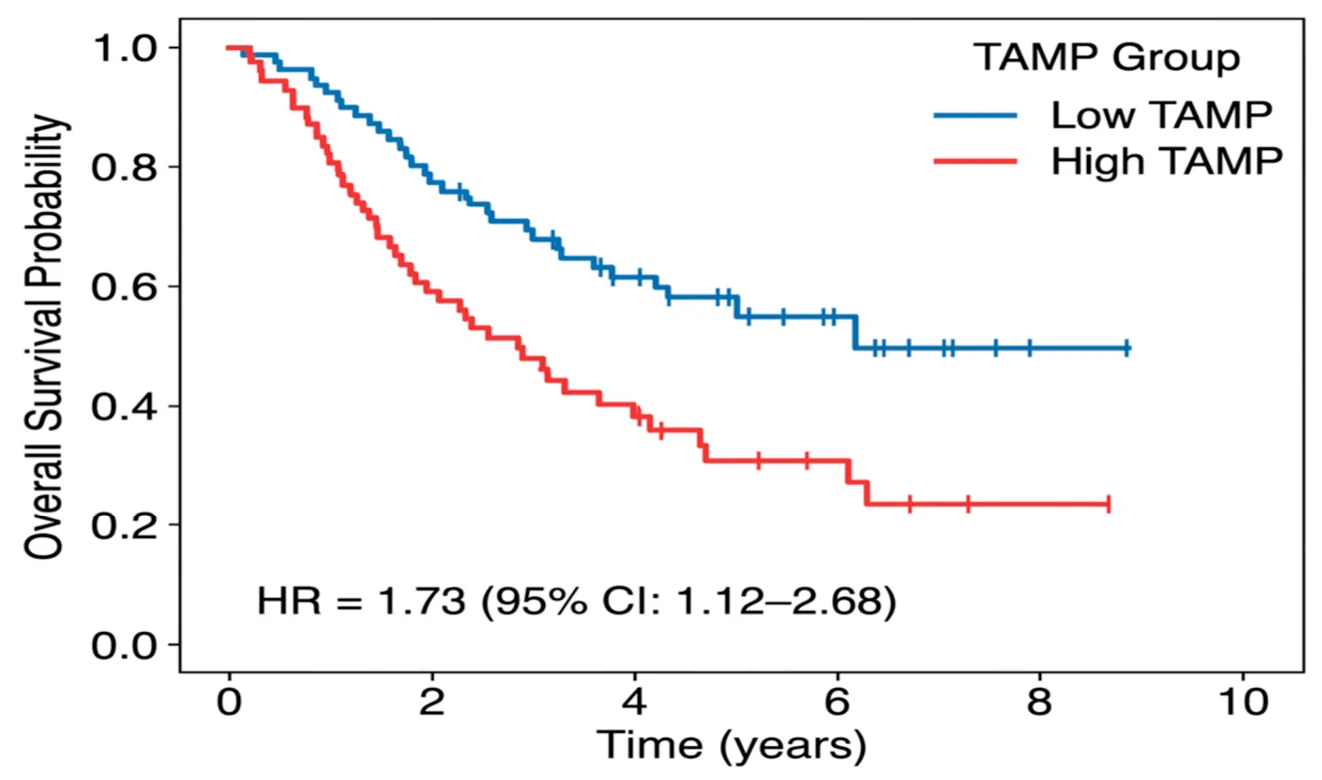
Kaplan–Meier overall survival curves stratified by tumor-area mitosis proportion (TAMP) in the TCGA-LUSC cohort. Patients were divided into high- and low-TAMP groups using the median TAMP value. The high-TAMP group showed significantly poorer overall survival than the low-TAMP group (log-rank *p* = 0.017). Univariate Cox proportional-hazards analysis yielded a hazard ratio of 1.73 (95% CI: 1.12–2.68) for the high-TAMP group relative to the low-TAMP group.

**Figure 3:**
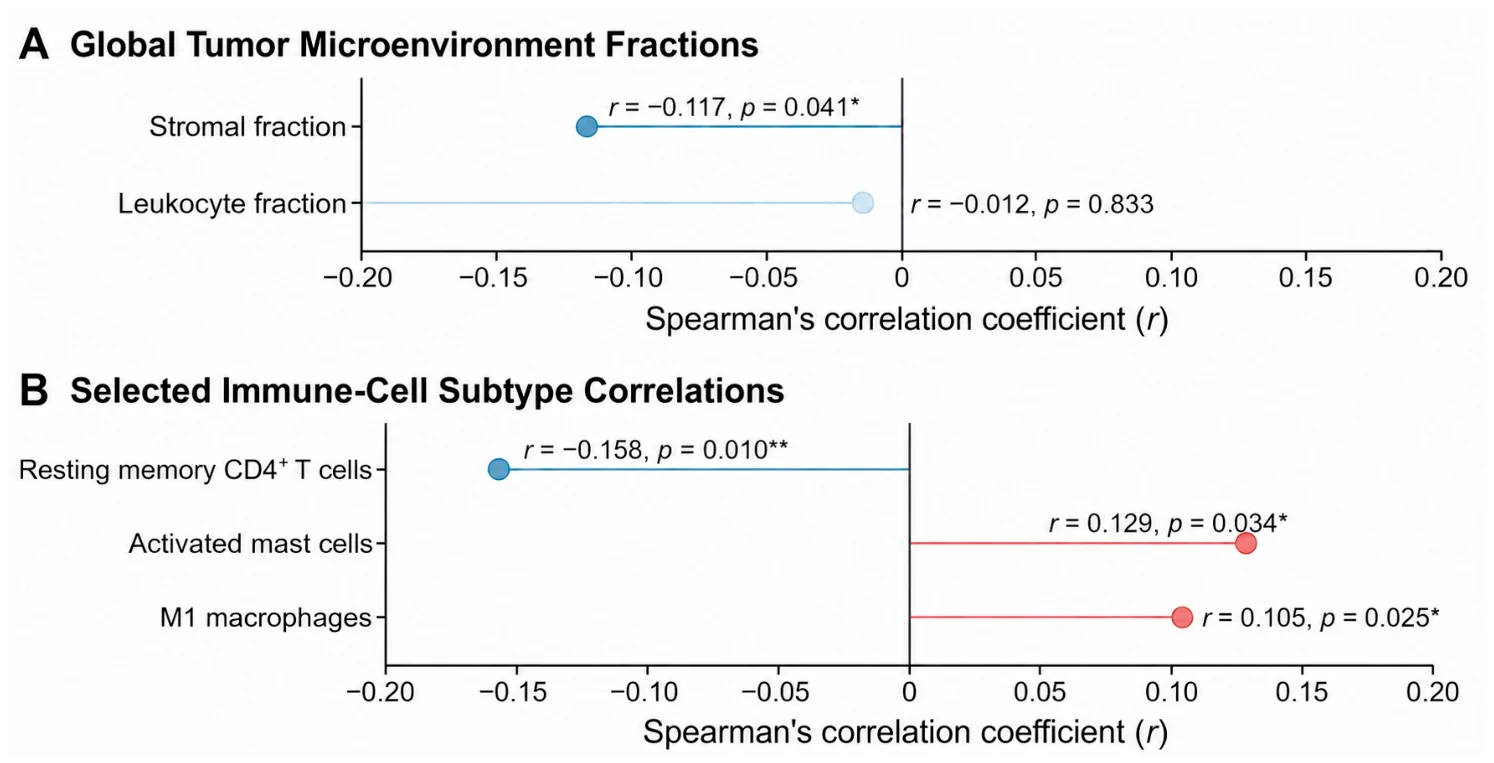
Exploratory associations between TAMP and selected components of the tumor microenvironment in the TCGA-LUSC cohort. TAMP was weakly negatively correlated with stromal fraction (ρ = −0.117, unadjusted *p* = 0.041) and resting memory CD4^+^ T cells (ρ = −0.158, unadjusted *p* = 0.010), while activated mast cells (ρ = 0.129, unadjusted *p* = 0.034) and M1 macrophages (ρ = 0.105, unadjusted *p* = 0.025) showed weak positive correlations. Reported *p*-values are nominal and were not adjusted for multiple testing. * *p* < 0.05; ** *p* < 0.01.

**Figure 4:**
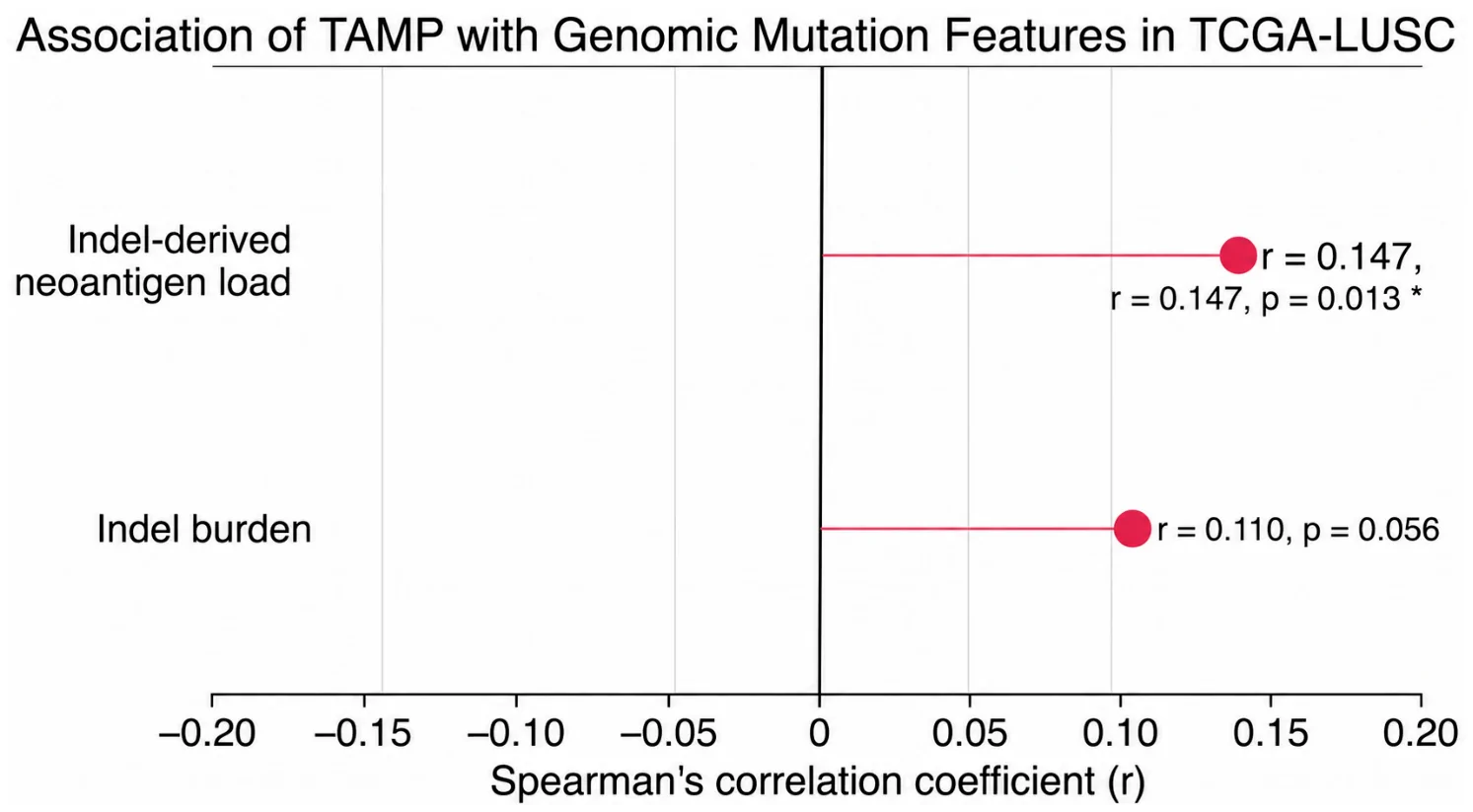
Exploratory associations between TAMP and selected genomic characteristics in TCGA-LUSC. TAMP showed a weak positive association with indel-derived neoantigen load (Spearman′s ρ = 0.147, unadjusted *p* = 0.013), whereas the association with overall indel burden was weaker (ρ = 0.110, unadjusted *p* = 0.056). Reported p-values are nominal and were not adjusted for multiple testing. * *p* < 0.05.

**Figure 5:**
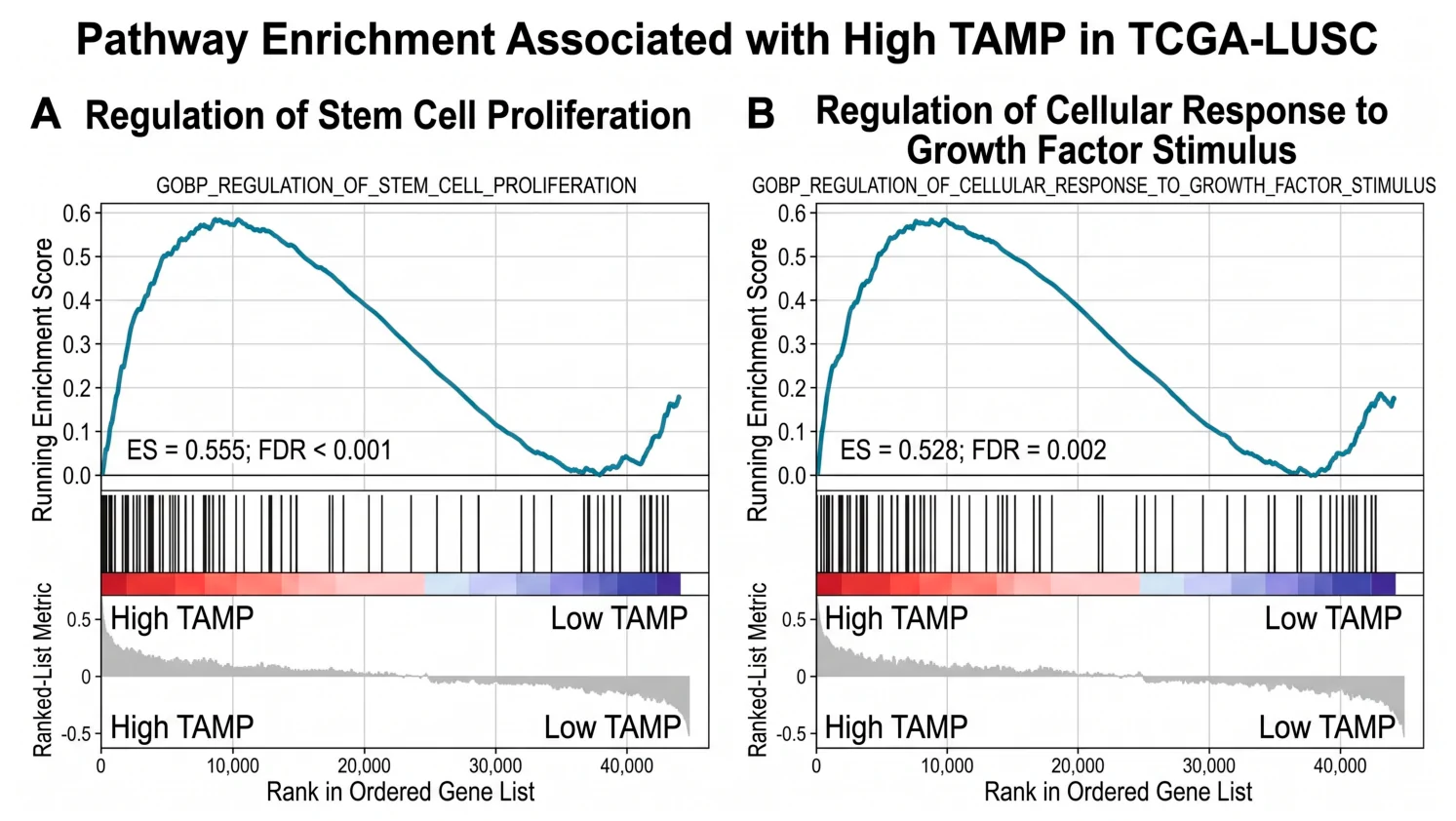
Pathway enrichment associated with TAMP in the TCGA-LUSC cohort. (**A**) GOBP_REGULATION_OF_STEM_CELL_PROLIFERATION was significantly enriched in tumors with high TAMP after false discovery rate (FDR) correction (ES = 0.555, FDR < 0.001). (**B**) GOBP_REGULATION_OF_CELLULAR_RESPONSE_TO_GROWTH_FACTOR_STIMULUS was also significantly enriched in tumors with high TAMP after FDR correction (ES = 0.528, FDR = 0.002). Both gene sets showed positive enrichment in tumors with elevated mitotic activity.

**Figure 6:**
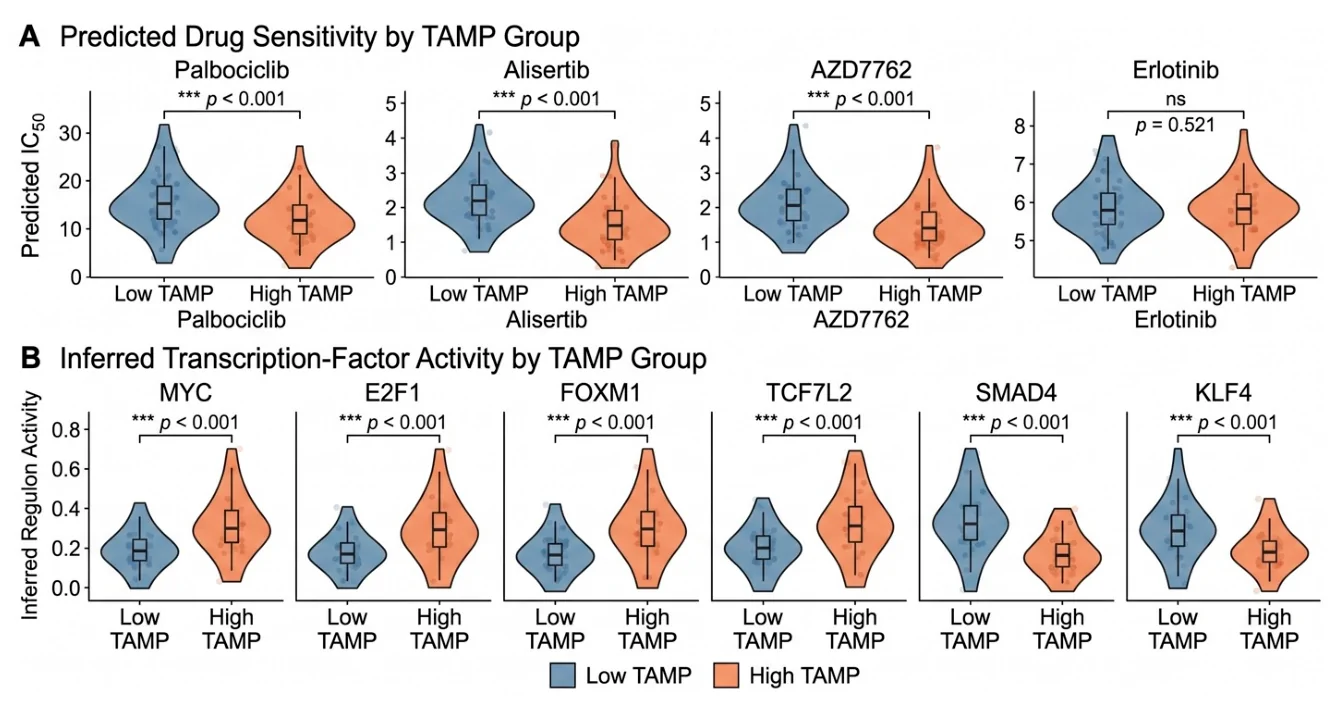
Predicted drug response and inferred transcription factor activity according to TAMP status in TCGA-LUSC. Tumors with high TAMP showed significantly lower predicted IC_50_ values for palbociclib, alisertib, and AZD7762 (*** *p* < 0.001), whereas erlotinib showed no significant difference (*p* = 0.521). MYC, E2F1, FOXM1, and TCF7L2 showed higher inferred activity in tumors with high TAMP, while SMAD4 and KLF4 showed lower inferred activity (*** *p* < 0.001).

**Table 1:** Cohort composition and data partitioning used for model development and evaluation.

Cohort	Role	Patients/Cases	WSIs	Patch Information	Evaluation
MIDOG	Source-domain development	150 cases	150	Source-domain patches extracted from annotated cases; internal patch-level test set *n* = 2000	Source-domain training/internal testing
TCGA-LUSC	Target-domain adaptation	478 patients	512	Approximately 3% mitosis-positive tumor patches	Initial 70%/15%/15% patch-level split; patient-level five-fold cross-validation; complementary patient-disjoint 20% WSI holdout

Note: Because non-mitotic tissue patches substantially outnumbered mitosis-positive patches, 50,000 non-mitotic patches were randomly sampled as source-domain training controls.

**Table 2:** Ablation analysis of transfer-learning components evaluated using patient-level stratified five-fold cross-validation.

Configuration	Mean AUC (95% CI)	Mean PR-AUC	Δ AUC vs. Full Model
Full pipeline (reference)	0.88 (0.86–0.90)	0.24	—
Without MIDOG pre-training (random initialization)	0.79 (0.76–0.82)	0.11	−0.09
Full fine-tuning (no selective freezing)	0.85 (0.82–0.87)	0.19	−0.03
Weighted BCE instead of Focal Loss	0.86 (0.83–0.88)	0.20	−0.02
Without training augmentation	0.86 (0.84–0.89)	0.21	−0.02

Note: All ablation experiments used identical patient-level stratified five-fold cross-validation folds. Δ AUC represents the difference in mean AUC relative to the complete pipeline.

**Table 3:** Univariable and multivariable Cox proportional-hazards analyses of overall survival.

Variable	Univariable HR (95% CI)	p-Value	Adjusted HR (95% CI)	p-Value
High versus low TAMP	1.73 (1.12–2.68)	0.014	1.62 (1.03–2.54)	0.036
Age, per year	1.02 (1.01–1.03)	0.004	1.02 (1.01–1.03)	0.006
Male versus female	1.28 (0.92–1.78)	0.150	1.22 (0.87–1.70)	0.250
Stage 2 versus 1	1.35 (0.85–2.15)	0.200	1.30 (0.81–2.08)	0.270
Stage 3 versus 1	2.10 (1.35–3.27)	0.001	2.02 (1.28–3.18)	0.003
Stage 4 versus 1	2.85 (1.55–5.24)	0.001	2.60 (1.39–4.86)	0.003

Abbreviations: CI, confidence interval; HR, hazard ratio; TAMP, tumor-area mitosis proportion. Stage 1, female sex, and low TAMP served as the reference categories.

## Data Availability

Data were analyzed in accordance with the access and use requirements of the corresponding source repositories.
